# CyanoPhyChe: A Database for Physico-Chemical Properties, Structure and Biochemical Pathway Information of Cyanobacterial Proteins

**DOI:** 10.1371/journal.pone.0049425

**Published:** 2012-11-21

**Authors:** P. V. Parvati Sai Arun, Ranjith Kumar Bakku, Mranu Subhashini, Pankaj Singh, N. Prakash Prabhu, Iwane Suzuki, Jogadhenu S. S. Prakash

**Affiliations:** 1 Department of Plant Sciences, School of Life Sciences, University of Hyderabad, Hyderabad, Andhra Pradesh, India; 2 Department of Biotechnology, School of Life Sciences, University of Hyderabad, Hyderabad, Andhra Pradesh, India; 3 Faculty of Life and Environmental Science, University of Tsukuba, Tsukuba, Japan; Massey University, New Zealand

## Abstract

CyanoPhyChe is a user friendly database that one can browse through for physico-chemical properties, structure and biochemical pathway information of cyanobacterial proteins. We downloaded all the protein sequences from the cyanobacterial genome database for calculating the physico-chemical properties, such as molecular weight, net charge of protein, isoelectric point, molar extinction coefficient, canonical variable for solubility, grand average hydropathy, aliphatic index, and number of charged residues. Based on the physico-chemical properties, we provide the polarity, structural stability and probability of a protein entering in to an inclusion body (PEPIB). We used the data generated on physico-chemical properties, structure and biochemical pathway information of all cyanobacterial proteins to construct CyanoPhyChe. The data can be used for optimizing methods of expression and characterization of cyanobacterial proteins. Moreover, the ‘Search’ and data export options provided will be useful for proteome analysis. Secondary structure was predicted for all the cyanobacterial proteins using PSIPRED tool and the data generated is made accessible to researchers working on cyanobacteria. In addition, external links are provided to biological databases such as PDB and KEGG for molecular structure and biochemical pathway information, respectively. External links are also provided to different cyanobacterial databases. CyanoPhyChe can be accessed from the following URL: http://bif.uohyd.ac.in/cpc.

## Introduction

As proteins mediate and control coordinated biochemical transformations and cellular processes that are central to activity of life forms, characterization of proteins provide insights into the structure and function of a cell [1], [2]. Pure form of a protein is needed for its characterization and can be extracted through expression and purification techniques. Instead of getting an active and soluble form of a protein, there are chances for a protein to enter into an inclusion body [3], [4]. Obtaining functionally active protein from an inclusion body requires denaturation of the protein, followed by refolding into its native form. This is a slow and difficult process which greatly reduces the net yield and activity of the protein [5]. Therefore, prevention of a protein to enter into inclusion body is better than resolving it. Solubility of a protein depends on its physico-chemical properties. For instance, length of a protein, composition and properties of amino acid residues in a protein influence its solubility [6]. Folding of an expressed protein also depends on the conditions employed during the process of expression and purification. It is possible to prevent the aggregation of a protein by providing suitable conditions based on its physico-chemical properties. The physico-chemical properties can be used to predict the nature of a protein and this information is useful for optimization of expression methods. These properties help to understand the native environment in which the protein will be in soluble and active form, thus aids the researchers in the characterization studies on the proteins of interest. In addition to the available traditional standard laboratory methods for determining physico-chemical properties of a protein, mathematical methods have also been in use for calculating the same based on primary sequence information [6]–[13]. Though different web based tools are available to determine physico-chemical properties of proteins [1], [14]–[17], it is time consuming and difficult task for a naïve user in choosing a tool among the available pool.

**Figure 1:**
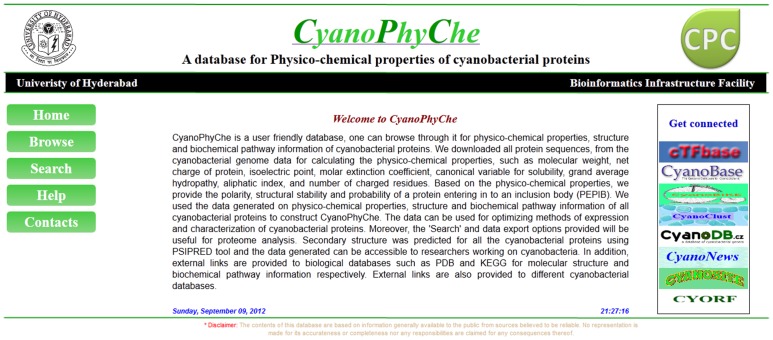
A snapshot of the homepage of CyanoPhyChe database. User can navigate for physico-chemical properties of any cyanobacterial protein by browsing through ‘Browse’ link located at the top left corner of the home page. Proteins with specific properties can be retrieved using ‘Search’ option. Links to external cyanobacterial databases are provided in the main page of the database.

Cyanobacteria are a group of oxygenic photosynthetic microorganisms that survived since life took its form on Earth. Because of their vital metabolic pathways and being contributors to global carbon and nitrogen budgets, they are one of the mostly studied microbes. At present, there are 38 cyanobacterial species for which total genome sequence information is available (http://genome.kazusa.or.jp/cyanobase). The available genome sequence information can be used to generate huge data, by applying bioinformatic, functional and comparative genomic approaches, which would address several questions related to the evolution, adaptation, physiology and biochemistry of cyanobacteria. Nevertheless, there is no database which can provide physico-chemical properties of cyanobacterial proteins. This motivated us to make a database on physico-chemical properties of cyanobacterial proteins using different tools and formulae which are scientifically proven to be accurate [6], [10], [13]–[16]. Thus, in this report we provide a user friendly database in which one can easily search for the properties of a cyanobacterial protein(s) in question and get preliminary understanding about it. We used the genome data from the database of cyanobacteria for calculating the physico-chemical properties of all cyanobacterial proteins and generated a database called ‘CyanoPhyChe’. Researchers can use the physico-chemical properties of any cyanobacterial protein that is available in the database for their research. The information provided in the database aids researchers for choosing optimal conditions for expression, purification and characterization of a cyanobacterial protein. Moreover, user can export the physicochemical properties, predicted secondary structure, amino acid sequence and amino acid composition of selected cyanobacterial proteins for further analysis. External links are provided to make a direct access to other cyanobacterial databases available on the internet.

**Figure 2:**
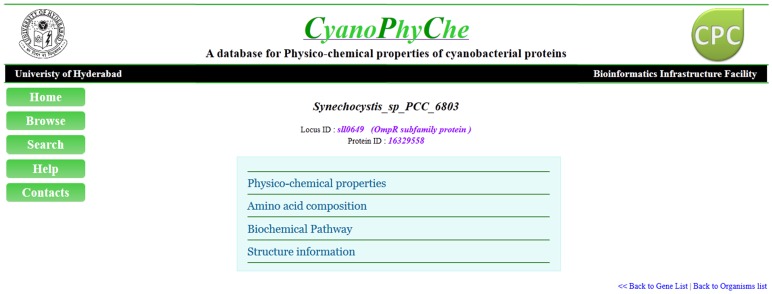
A snapshot of an access page. This page contains a menu with physico-chemical properties, amino acid composition, biochemical pathway and structure information.

## Materials and Methods

### Protein Sequence Data for Calculating Physico-chemical Properties

Total 38 files with extension.faa, each containing all protein sequences of a cyanobacterial species, were downloaded from NCBI (ftp://ftp.ncbi.nih.gov/genomes/Bacteria). A code was developed in PERL to separate all the protein sequences of a ‘.faa’ file into multiple FASTA files, each with an individual protein sequence. This primary seed data is used for calculating protein properties. Seed data contained total 1,26,610 proteins, covering 38 cyanobacteria.

**Figure 3:**
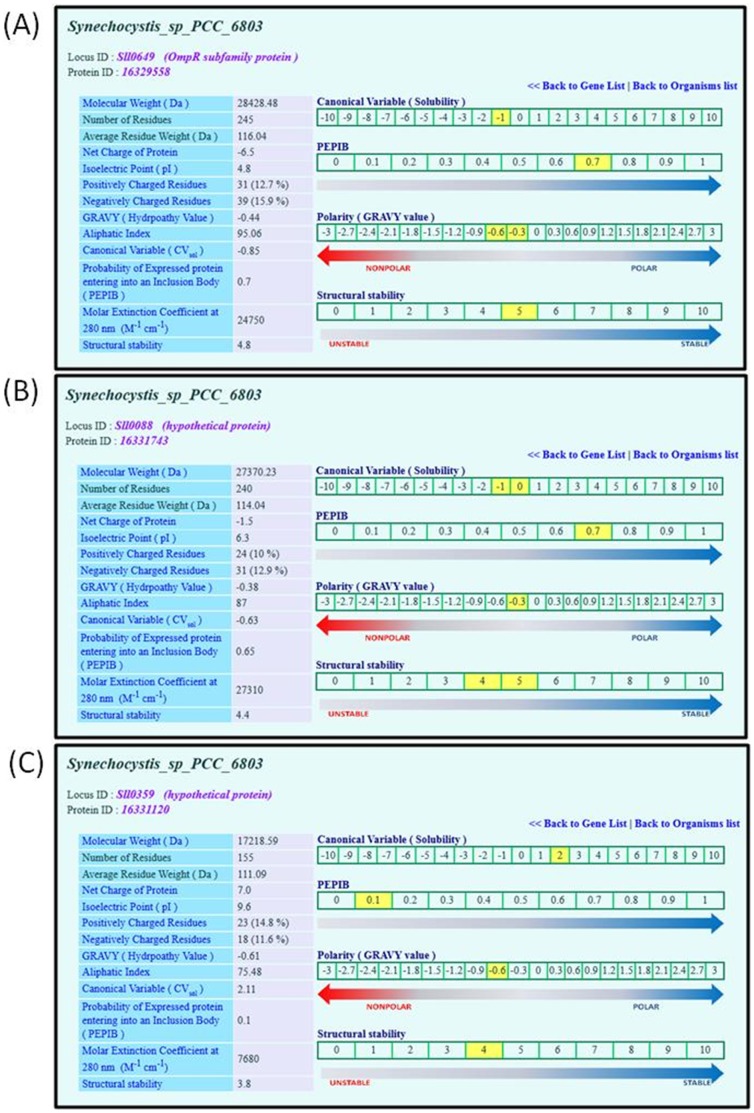
A snapshot of physico-chemical properties of the selected proteins. Physico-chemical properties of (A) Sll0649, (B) Sll0088 and (C) Sll0359 proteins from *Synechocystis* sp. PCC6803. Based on the presented physico-chemical properties of, solubility, probability of the protein entering into an inclusion body (PEPIB), polarity and structural stability were calculated and indicated on a point scale for better visualization of its nature.

Certain physico-chemical properties were calculated using PEPSTATS tool, which is available in EMBOSS package, installed in a computer using Linux mint-12 operating system (http://www.ebi.ac.uk/Tools/emboss/pepinfo/). PEPSTATS provides molecular weight, number of residues, isoelectric point (pI), molar extinction coefficient and amino acid composition of a protein [14]. The output file generated by PEPSTATS was used as a secondary seed data for calculating other properties like, aliphatic index (AI), GRAVY, canonical variable for solubility (*CV*_sol_) and probability of expressed protein entering into an inclusion body (PEPIB).

**Figure 4:**
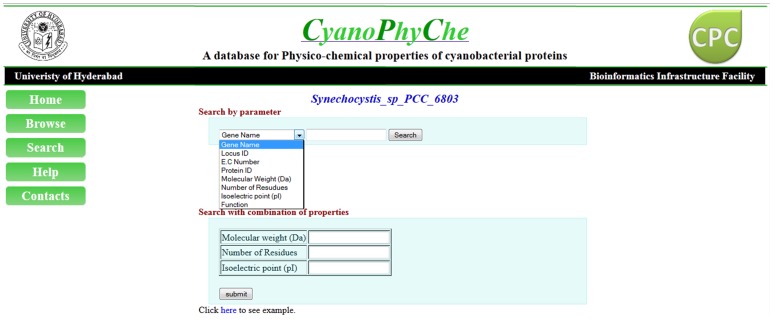
A snapshot showing the CyanoPhyChe ‘Search’ page. A dropdown menu provided in the search page with various parameters can be used for retrieving cyanobacterial proteins in the database. Proteins of a cyanobacterium can be retrieved by gene name, locus ID, E.C. number and protein ID, and properties like molecular weight, number of residues, and pI value. User can also retrieve the cyanobacterial protein(s) by searching the database using combination of two or more properties listed under “Search with combination of properties” menu.

#### Aliphatic index and structural stability

Stability of a protein can be calculated using aliphatic index. We used the formula that was developed for determining aliphatic index [10], [18]. The aliphatic index values of cyanobacterial proteins were normalized between 0 and 10 to predict the structural stability.

#### Grand average value of hydropathy (GRAVY)

An empirical formula for calculating hydropathy value for a protein was developed by Kyte and Doolittle (1982), wherein the hydrophilic and hydrophobic properties of each amino acid side chain in a protein are taken into consideration [13]. Positive hydropathy value is indicative of a polar protein and negative value indicates a non-polar protein.

#### Canonical variable for solubility (CVsol) and PEPIB

A mathematical formula, based on the amino acid composition and their properties, was derived to predict the solubility of a protein and its probability to enter into an inclusion body [6]. The solubility or insolubility of a protein can be predicted from the canonical variable, which is a composite parameter of cysteine fraction, proline fraction, turn forming residue fraction, approximate charge average, number of residues and hydrophilicity, according to Wilkinson and Harrison model [6]. The formula for calculating the canonical variable is given below.
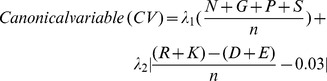
where,

*n* = number of amino acids in protein

*N*, *G, P*, *S* = number of Asn, Gly, Pro & Ser residues respectively.

*R*, *K*, *D*, *E* = number of Arg, Lys, Asp & Glu residues.

*λ*_1_ and *λ*_2_ =  Coefficients (15.43 and −29.56 respectively) and

Canonical variable for solubility (CV_sol_) = (CV–CV′)

*Canonical variable for solubility* 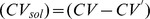
where, *CV′* = 1.71.

*Probability of solubility or insolubility* = 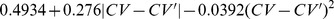.

The formula for calculating probability of solubility or insolubility for any protein when expressed in *E. coli* was further evaluated by Harrison 2000 [5]. Also, a detailed description of canonical variable and its usage has been given by Koschorreck et al. 2005 [19]. Based on the sign of *CV*_sol_ value PEPIB was determined. If *CV*_sol_ value is positive, the above equation provides the probability of insolubility, which is considered as PEPIB. If *CV*_sol_ value is negative, the equation provides the probability of solubility [5]. PEPIB of these proteins were calculated by converting their probability of solubility in to the probability of insolubility, considering the fact that the sum of probability of solubility and insolubility must be one.

### Secondary Structure Prediction

Secondary structure prediction was done using PSIPRED tool [15]. UNIREF90 database was used as target for PSI-Blast (ftp://ftp.ebi.ac.uk/pub/databases/uniprot/uniref/). A BASH shell program was designed to automate this tool for predicting secondary structure for cyanobacterial proteins.

### Design of CyanoPhyChe Database and its Accessibility

The CyanoPhyChe database was developed using MySQL database management system (MySQL Version 5.1.4.1 and php MyAdmin 3.2.4). Web interface was designed using HTML, Java script and PHP to retrieve and visualize the data. PDB IDs and biochemical pathway IDs were extracted from NCBI (ftp://ftp.ncbi.nih.gov/genomes/Bacteria) and KEGG (http://www.genome.jp/kegg/) databases respectively for all cyanobacterial proteins. The pathway IDs were provided with links using HTML and PHP scripting. CyanoPhyChe can be accessed from a local web server located in Bioinformatics Infrastructure Facility at School of life Sciences, University of Hyderabad, India using the following URL: http://bif.uohyd.ac.in/cpc.

## Results and Discussion

### Description of CyanoPhyChe

The web interface contains ‘Home’, ‘Browse’, ‘Search’, ‘Help’ and ‘Contact’ links on the top left corner of the index page for browsing the database (Figure 1). A brief introduction about CyanoPhyChe is given in the home page. The physico-chemical properties, structural and biochemical pathway information of any cyanobacterial protein can be accessed either by ‘Browse’ or ‘Search’ link. Further, the webpage contains external links to other cyanobacterial databases such as cyanobase, cyanoclust, cyanoDB.cz and cyanoBIKE. This will assist the user to get a direct access to other related databases for additional information on cyanobacteria.

### Browsing the Database

As the user goes through the ‘Browse’ option, the list of cyanobacteria is displayed. Name of each cyanobacterium is further linked to a table that lists all the proteins coded by its genome, along with their protein ID, locus ID, gene name and function. Upon a single click on any protein, an access page appears that displays links to physico-chemical properties, amino acid composition, biochemical pathway and structure information of the selected protein (Figure 2). User can select more than one protein from the protein-list of a cyanobacterium and export the data in CSV format. In addition, the user can also download the secondary structure, protein sequence and amino acid composition of the selected proteins.

#### Physico-chemical properties

Browsing through the link ‘Physico-chemical properties’ leads to a table containing physico-chemical properties of the protein in question. In addition, four different property scales with gradient-colored arrows are provided to aid the user predict the protein’s nature, such as solubility, polarity and structural stability, at a glimpse (Figure 3). Scales are provided for the canonical variable (*CV*_sol_), probability of an expressed protein entering into an inclusion body (PEPIB), GRAVY and structural stability (Figure 3). When the user browses through “Physico-chemical properties” of a protein for visualizing its properties, canonical variable for solubility (*CV*_sol_), GRAVY value, PEPIB value, and structural stability are indicated on their respective scales (Figure 3). Property values of a selected protein are highlighted with blinking on the respective scales. Figure 3 shows the determined physico-chemical properties of three selected proteins, Sll0649, Sll0088, and Sll0359 from *Synechocystis* sp. PCC 6803. PEPIB values calculated for Sll0649 and Sll0088 are 0.7 and 0.65, respectively. These values indicate that there is a high chance for these proteins to enter into inclusion body during their heterologous expression. To validate our predictions, we expressed these proteins to verify whether the calculated properties of the proteins can be relied upon and be considered for optimizing the conditions of expression, purification and characterization. Upon expression, most of the Sll0649 and Sll0088 were found in the inclusion body and a little was observed in the soluble fraction (Figure S1A and S1B). PEPIB value of Sll0359 protein is 0.1, hence it is predicted to be a soluble protein during its heterologous expression. As predicted, this protein was appeared in soluble fraction upon expression in *E. coli* (Figure S1C). These results are well in agreement with the calculated properties. The molar extinction coefficients calculated for the above proteins are 24750, 27310 and 7680 M^−1^ cm^−1^, respectively This values can be used to determine the concentration of purified proteins by measuring their absorbance at 280 nm. Temperature is one of the factors that affect the structure of a protein. Studies show that there is a positive correlation between the structural stability and aliphatic amino acid content of proteins [10], [18]. The aliphatic index values of Sll0649, Sll0088 and Sll0359 are 95.1, 87.0 and 75.5. These values give the structural stability value of 4.8, 4.4 and 3.8 for these three proteins, respectively. This indicates that these proteins are moderately stable.

The isoelectric point of a protein is an important property, because protein is least soluble at the pH near this point. There is a significant relation between pI of a protein and pH of the buffer being used for crystallization. The buffer pH equals to or very near to the pI value of a protein offers a reasonable probability of yielding crystals [20]. The CyanoPhyChe database provides pI value for all the cyanobacterial proteins. Calculated pI of a cyanobacterial protein can be used to select a suitable buffer condition for its crystallization.

#### Amino acid composition and structure information

Amino acid composition and structural information of a cyanobacterial protein in question can be visualized by browsing through the links ‘Amino acid composition’ and ‘Structure information’, respectively. The ‘Structure information’ link navigates to the page containing predicted-secondary structure. In addition, an external link is provided to PDB database (http://www.rcsb.org/pdb/home/home.do) for viewing the 3D structure, when it is available. If the structure is not available for a protein, then the user can look for homologous proteins whose structures are available in the PDB database, by clicking on the “BLAST” tab for building a homology model.

#### Biochemical pathway in which a protein is involved

If a protein is known to be involved in any biochemical pathway, an external link to KEGG database is provided under the pathway name and ID that navigates to and displays the pathway in which it is involved (http://www.genome.jp/kegg/pathway.html).

### Search Options in the CyanoPhyChe Database

Search page contains the list of cyanobacteria. User can choose one or more organisms from the list for finding proteins with desired properties. The selection leads to a query page, where search option to retrieve a protein or a group of proteins with specific properties from the selected cyanobacteria have been provided (Figure 4). User can search the database using identifiers, such as gene name, locus ID, E.C. number, function and protein ID. The search window allows the user to enter a string of values for the selected identifier to search for different proteins among the selected organisms. The user can also search for the proteins based on the properties like molecular weight, number of residues and pI value.

An option is provided to search and display proteins of a cyanobacterium that fall within a given range of a property. For instance, searching the database to retrieve the proteins with pI values ranging from 8 to 9 in *Synechocystis* sp. PCC6803, displays the list of proteins fall within this range. User can also retrieve the cyanobacterial protein(s) by searching the database using a specific property value or combination of two or more properties listed under “Search with combination of properties” menu. This option is more useful for retrieving proteins with different combination of properties, such as molecular weight, number of residues and isoelectric point. Physicochemical properties of the listed proteins from a single or multiple cyanobacterial species, using above search criteria can be exported in the CSV format. Additionally, the user can also export the secondary structure, protein sequence and amino acid composition of the resulted proteins. These features of the database will be more useful to the researchers working on proteome analysis of any cyanobacterium.

### Conclusions

In summary, the database CyanoPhyChe is a collection of the calculated physico-chemical properties, solubility, and probability of an expressed protein entering into an inclusion body, structural stability, polarity and secondary structure of all cyanobacterial proteins. External links to PDB structure and KEGG pathway are provided in the database. Search option facilitates the retrieval of proteins of a particular cyanobacterium with specific property or combination of more than one property. The database also allows the user to export the retrieved data and encourages to use it for comparative studies. The data provided in the database can be used by the researchers, who are working on the cyanobacterial proteins for optimizing the methods employed for expression, purification, and characterization. The database is also useful for interpreting the results obtained from proteome analysis of cyanobacteria. CyanoPhyChe will be further updated with additional information on cellular localization of cyanobacterial proteins and physico-chemical properties of the proteins encoded by the plasmid-DNA of cyanobacteria in the upcoming versions. Further, the database will be constantly updated and curated by the authors, as and when new information is reported in the literature or communicated by the users.

## Supporting Information

Figure S1 **SDS-PAGE analyses of soluble and insoluble fractions of** ***E. coli*** **expressing the** ***Synechocystis*** **sp. PCC6803 proteins.** Solubility of (A) Sll0649, (B) Sll0088 and (C) Sll0359 proteins. 0.4 mM IPTG (final concentration) was added to *E. coli* cells for inducing expression. The cells were harvested for separation of soluble and insoluble protein fractions, 2 hours after induced expression as described above. IS, Insoluble fraction; S, soluble fraction. Expressed protein bands are shown by open arrows.
(DOCX)
