# Supplementary material for: CyanoPhyChe: A Database for Physico-Chemical Properties, Structure and Biochemical Pathway Information of Cyanobacterial Proteins
Source: PLoS One. 2012 Nov 21;7(11):e49425. doi: 10.1371/journal.pone.0049425 (PMC3504015; doi:10.1371/journal.pone.0049425)
Supplement: Figure S1 — SDS-PAGE analyses of soluble and insoluble fractions of E. coli expressing the Synechocystis sp. PCC6803 proteins. Solubility of (A) Sll0649, (B) Sll0088 and (C) Sll0359 proteins. 0.4 mM IPTG (final concentration) was added to E. coli cells for inducing expression. The cells were harvested for separation of soluble and insoluble protein fractions, 2 hours after induced expression as described above. IS, Insoluble fraction; S, soluble fraction. Expressed protein bands are shown by open arrows. (DOCX) [file pone.0049425.s001.docx]

**Figure S1:** SDS-PAGE analyses of soluble and insoluble fractions of *E. coli* expressing the *Synechocystis* sp. PCC6803 proteins. Solubility of (A) Sll0649, (B) Sll0088 and (C) Sll0359 proteins*.* 0.4 mM IPTG (final concentration) was added to *E. coli* cells for inducing expression. The cells were harvested for separation of soluble and insoluble protein fractions, 2 hours after induced expression as described above. IS, Insoluble fraction; S, soluble fraction. Expressed protein bands are shown by open arrows.

**Supplementary Protocol S1**

**Heterologous expression of *Synechocystis* proteins in *E. coli*:**

Open reading frames coding for Sll0649, Sll0088 and Sll0359 were amplified, by PCR, with the primer sets Sll0649-F: 5' -GAC TCA TAT GTG GGG GAA CAG GAC TGA A -3' and Sll0649-R: 5' - GCT GAA TTC TTA ATC AGG GTC TTC AAA CTT AT-3', Sll0088-F: 5'-GAC TCA TAT GGG GGT TGT CCT TTC AGT T -3' and Sll0088-R: 5' -GCT GAA TTC TTA GTT CGG GGT TTT AGA CTG G -3' and Sll0359-F: 5' -GAC TCA TAT GCC AAA CGC CTC CAC CGC-3' and Sll0359-R: 5' -GCT GAA TTC TTA TAC TTC CTC TTC GTC ATC G -3'. The amplified ORFs were eluted from the gel and were inserted into pET-28a(+) at suitable restriction enzyme sites to generate pET-Sll0649, pET-Sll0088 and pET-Sll0359. The N-terminally His-tagged proteins were expressed in BL21(DE3)pLysS, which had been transformed with the above DNA constructs. The expression of each protein was induced by addition of 0.4 mM (final concentration) IPTG. Bacterial cells were collected by centrifugation at 10 000 g for 10 min and pelleted cells were disrupted with a sonic oscillator (model, UV2070; probe, MS-72; Bandelin Electronic) operated for 10 min at 50% power, with 1 min pulse interval, in 100 mM Tris/HCl (pH 8.0) and 200 mM NaCl. Soluble supernatant and insoluble precipitates were separated by centrifugation at 20,000 g for 20 min at 4^o^C. Insoluble fractions were suspended in the same buffer which contained a 0.5% Triton -X 100. the soluble and insoluble proteins were resolved on 12% SDS-PAGE.
